# Terahertz charge transport dynamics in 3D graphene networks with localization and band regimes

**DOI:** 10.1039/d2na00844k

**Published:** 2023-05-02

**Authors:** Prabhat Kumar, Martin Šilhavík, Manas R. Parida, Hynek Němec, Jiří Červenka, Petr Kužel

**Affiliations:** a FZU – Institute of Physics of the Czech Academy of Sciences Na Slovance 2 Prague 8 18221 Czech Republic kuzelp@fzu.cz

## Abstract

Terahertz steady-state and time-resolved conductivity and permittivity spectra were measured in 3D graphene networks assembled in free-standing covalently cross-linked graphene aerogels. Investigation of a transition between reduced-graphene oxide and graphene controlled by means of high-temperature annealing allowed us to elucidate the role of defects in the charge carrier transport in the materials. The THz spectra reveal increasing conductivity and decreasing permittivity with frequency. This contrasts with the Drude- or Lorentz-like conductivity typically observed in various 2D graphene samples, suggesting a significant contribution of a relaxational mechanism to the conductivity in 3D graphene percolated networks. The charge transport in the graphene aerogels exhibits an interplay between the carrier hopping among localized states and a Drude contribution of conduction-band carriers. Upon photoexcitation, carriers are injected into the conduction band and their dynamics reveals picosecond lifetime and femtosecond dephasing time. Our findings provide important insight into the charge transport in complex graphene structures.

## Abstract 2

3D Graphene aerogels add high porosity and elasticity to the optoelectronics of the graphene family materials. Their charge carrier transport is governed by an interplay of the hopping and Drude-like intraband conductivity.

## Graphical abstract

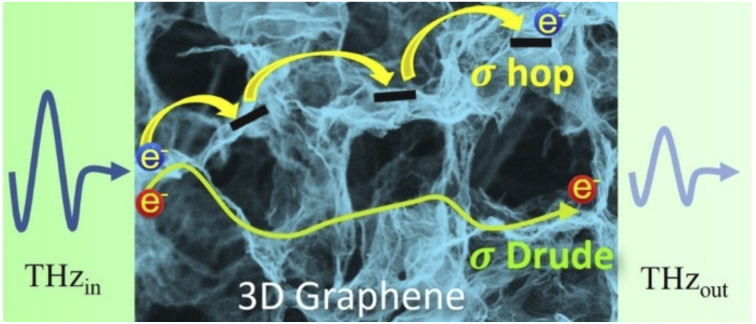

## Introduction

Graphene has emerged as an outstanding two-dimensional (2D) material due to its unique optical and electronic properties such as high electron mobility,^1,2^ charge-density tunability,^3^ wideband absorption,^3,4^ and highly nonlinear terahertz (THz) response,^5^ tunable terahertz photoconductivity,^6^*etc.* These highly-demanded properties open up new opportunities for graphene in various high-performance devices, such as fast field-effect transistors,^7^ far-infrared detectors,^8,9^ plasmonic devices,^10^ harmonic generators and frequency converters,^11^ optical electric field modulator.^12^ For such devices, it is of primary importance to accurately understand graphene's optical and electronic properties in the THz regime.

Single-layer graphene exhibits optical absorption in the visible to mid-IR range close to the well-known theoretical value of *e*_0_^2^/4*ħ*, due to the interband carrier transitions.^4,13^ However, in the THz region, doped graphene rather exhibits a Drude conductivity behavior^14–16^ owing to dominating intraband transitions. A plasmonic conductivity peak is also frequently observed in the THz range for epitaxial graphene.^14,15,17–19^ In multilayer graphene, THz wave absorption strongly depends on the misorientation angles and stacking arrangements between the graphene layers;^20^ the Drude conductivity then leads to a decrease in THz transmission in multilayer systems.^21^ Recently, a conductivity peak due to van Hove singularity at 2.7 THz on top of a Drude-like response observed in bilayer graphene was attributed to commensurate twisting of graphene layers.^22^ Briefly, multiple physical phenomena can modulate the electronic coupling between graphene layers, suggesting exciting new opportunities for tuning the THz response in more complex three-dimensional (3D) graphene structures. In contrast to extensive efforts on the graphene in a 2D form, optical studies of 3D graphene structures in the THz range are still limited^23,24^ and a deeper insight into the charge transport has not been reported yet.

In recent years, thanks to the development of graphene synthesis, 3D graphene networks assembled in free-standing covalently cross-linked graphene aerogel (GA) structures that maintain the characteristic properties of single-layer/multilayer graphene have been realized.^25,26^ Compared with traditional 2D graphenes, GAs provide high porosity and large effective surface area; GAs are also lightweight, elastic, highly conductive, and mechanically robust, making them promising for various applications, *e.g.*, strain and pressure sensing.^27^ However, THz optoelectronic properties of GAs have not been explored in detail yet.

In this work, we report experimental observations of the THz conductivity and permittivity in graphene assembled in free-standing covalently cross-linked GA. The experiments reveal an interplay between the Drude and hopping conductivity, which is significantly different from the transport observed in 2D graphene layers.^14–19,28^ We investigate the role of defects in GA on the character of the charge carrier transport by controlling the transition between reduced-graphene oxide and graphene using high-temperature annealing. Ultrafast THz photoconductivity measurements in high-temperature annealed samples reveal a photoinduced Drude-like absorption process with a picosecond characteristic decay.

## Experimental section

**Fig. 1:**
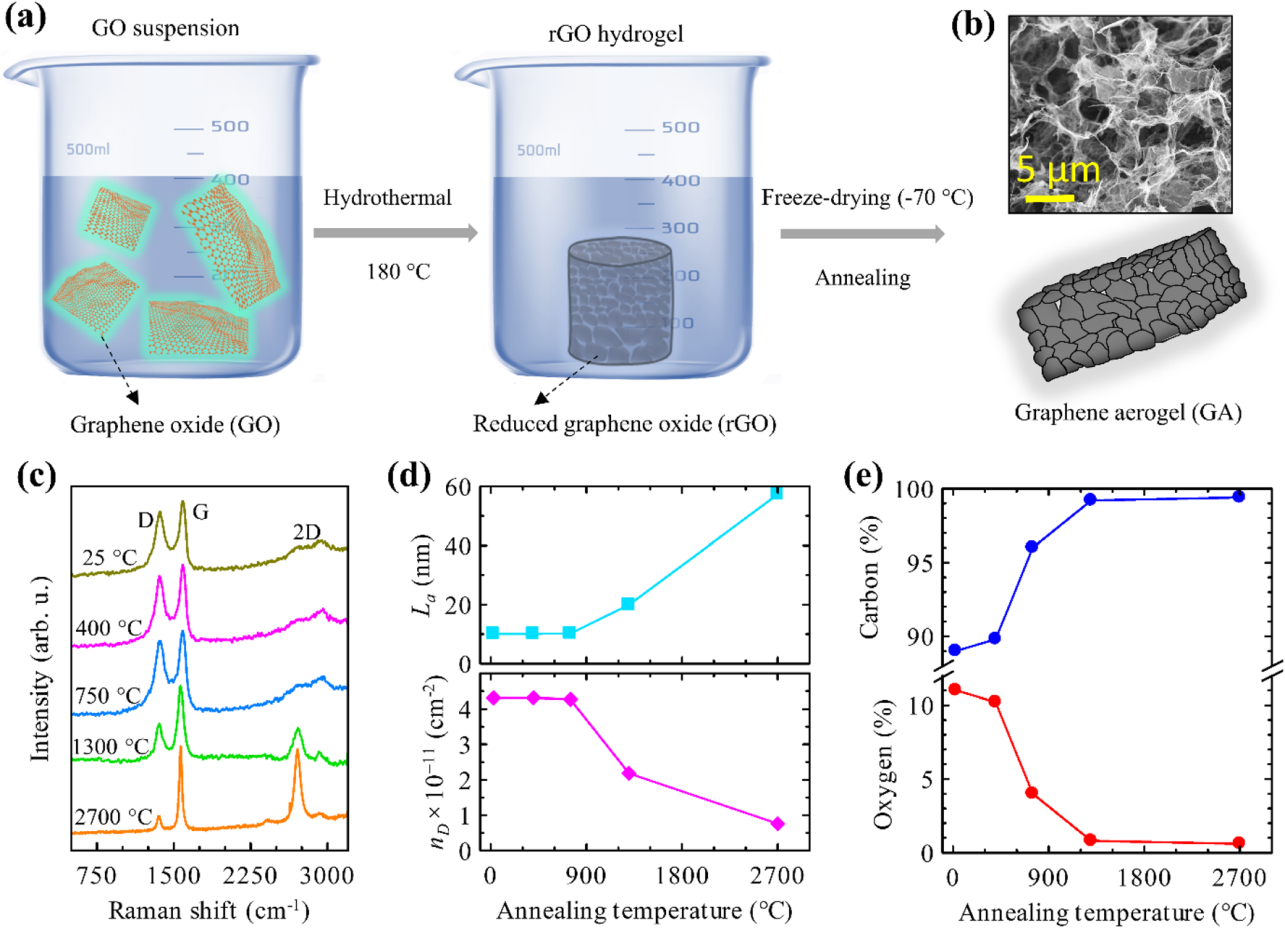
(a) Schematic for the synthesis of the graphene aerogels. (b) Scanning electron micrograph of GA annealed at 2700 °C. (c) Raman spectra. (d) Crystallite size (*L*_a_) and density of defects (*n*_D_) as calculated according to the methods by Cançado *et al.*^32,33^ (e) XPS compositional analysis of GAs annealed at different temperatures.

A scheme of the fabrication process is depicted in Fig. 1a. 60 mg of graphene oxide (GO) in powder (XFNANO company) was mixed with 30 ml of deionized water. GO solution was delicately blended and then immersed in an ultrasonic bath (sonication for 60 minutes at 30–40 °C). 50 ml of GO mixture was transferred into a Teflon-lined hydrothermal stainless-steel autoclave and annealed at 180 °C for 6 h. After the hydrothermal process, a chemically reduced and self-assembled 3D hydrogel structure was obtained. The hydrogel was washed with deionized water multiple times to remove the residuals and freeze-dried at −70 °C in vacuum (2 × 10^−1^ mbar) for 16 h to get a cylindrical sample of stable reduced graphene oxide (rGO) aerogel. The obtained rGO aerogel was kept at room temperature 25 °C and subsequently annealed in vacuum (2.3 × 10^−4^ mbar) at temperatures of 400, 750, 1300 and 2700 °C for 30 minutes. The samples were prepared with the initial thickness of 3–4 mm and their dc conductivity was determined at this stage. In order to obtain at least partially transparent samples for THz experiments, they were subsequently thinned down to various thicknesses (0.58 mm for the non-annealed sample, and 0.58, 0.47, 0.26, and 0.24 mm for the samples annealed at 400 °C, 750 °C, 1300 °C and 2700 °C, respectively).

Scanning electron microscopy (TESCAN MAIA3) was used to characterize the morphology of the samples. Furthermore, X-ray photoelectron spectroscopy (XPS Kratos Analytical Ltd) was employed to check the elemental composition and Raman spectroscopy (Renishaw inVia setup using a 442 nm laser) was used to assess the crystallinity and crystallite size of the samples. Dc conductivity measurements were performed by linear four-point probe method by slightly pressing the sample clamped between two glass slides on the top of predeposited electrodes (≤5% strain) to achieve a good electrical contact.

Femtosecond oscillator-based time-domain THz spectroscopy setup (using a photo-switch emitter TeraSED and an electro-optic gated detection scheme with 1 mm thick ZnTe)^29^ was used to measure the THz waveforms transmitted through the samples. The complex refractive index *N* of the samples was calculated from the transmission spectra including the Gouy shift correction.^29^ Subsequently, spectra of the complex permittivity *ε* = *N*^2^ and conductivity *σ* = −*iωε*_0_(*ε* − 1) were evaluated. In this work, we display real parts of *ε* and *σ* because they show the most pertinent spectral features. Low-temperature measurements were performed in an optical cryostat (Oxford, Optistat) with mylar windows. The thicknesses of the samples were measured separately using an Olympus optical microscope and used for the determination of their THz refractive indices.^30^

The optical pump – THz probe experiments in a collinear arrangement were carried out at room temperature using a Ti:sapphire ultrafast amplified laser system (Spitfire ACE, central wavelength 800 nm, 5 kHz repetition rate, 40 fs pulse length). The laser fundamental wavelength was used for the sample photoexcitation and also for the generation and electro-optic gated detection of the THz probing pulse in a pair of 1 mm thick (110) oriented ZnTe crystals. The sample was attached to a metallic aperture with an inner diameter of 3 mm. The pump beam was spatially expanded in order to excite the exposed part of the sample quasi-homogeneously. Samples thinned to ∼100 μm were completely opaque for the pump beam. This means that the effective optical absorption coefficient *α* of GA was larger than 25 mm^−1^. In this case, it can be shown^31^ that the THz photoconductivity spectrum (at a pump-probe delay *τ*_p_) can be evaluated using the following formula:1
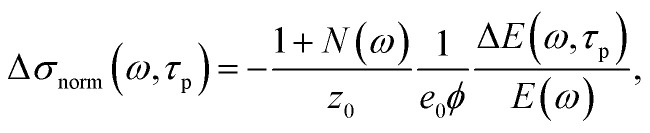
where Δ*σ*_norm_ is the photoconductivity of the aerogel sample per single unit charge, Δ*E* is the measured transient (differential) transmitted THz spectrum upon the sample photoexcitation with a photon fluence *ϕ*, *E* is the reference transmitted THz spectrum (without photoexcitation), *z*_0_ is the vacuum wave impedance, *e*_0_ is the unit charge, and *N* is the measured complex refractive index of the GA sample in equilibrium. The formula is deduced from the case (B1) of Fig. 4 in ref. 31, which is a pertinent approximation since the entire excitation power is absorbed in the sample.

## Results

Using the hydrothermal synthesis followed by freeze-drying and high-temperature annealing, we “engineered” cross-linked graphene networks in GA with the same morphology but with different amounts of structural defects. The morphology and Raman spectroscopy analysis of the GA samples are depicted in Fig. 1b–d. One observes a well interconnected 3D porous network in the GA (Fig. 1b). The sizes of the pores form a broad distribution ranging from sub-micrometers to several micrometers. The walls of the pores consist of ultrathin layers of graphene sheets stacked together. The covalent cross-linking of the graphene sheets has been confirmed by tensile and compressive mechanical testing in our previous study.^25^

Raman spectra of the non-annealed samples show two intense peaks at 1369 and 1596 cm^−1^, corresponding to the D and G bands of rGO. In contrast, GA samples annealed at 2700 °C depict an additional 2D peak at 2720 cm^−1^ and a significant reduction in the D peak intensity. This demonstrates that the annealing was efficient in improving the crystallinity and removing oxygen functional groups from the GA, facilitating a transition from rGO to graphene.

We evaluated the crystallite domain size (*L*_a_) and the density of defects (*n*_D_) in the samples from the Raman peak intensity ratio *I*(D)/*I*(G),^32,33^ see Fig. 1d. No noticeable change is observed in *L*_a_ and *n*_D_ upon annealing up to 750 °C. However, as the annealing temperature is raised to 1300 °C, a clear increase in the crystallite size along with the reduction of the defect concentration occurs. At this temperature, most of the oxygen functional groups are unstable and desorb from the rGO,^34–37^ as corroborated with the XPS compositional analysis in Fig. 1e. This trend continues with the sample annealed at 2700 °C: the resulting GA features a change of *L*_a_ and *n*_D_ by a factor of ∼6 compared to the non-annealed GA.

**Fig. 2:**
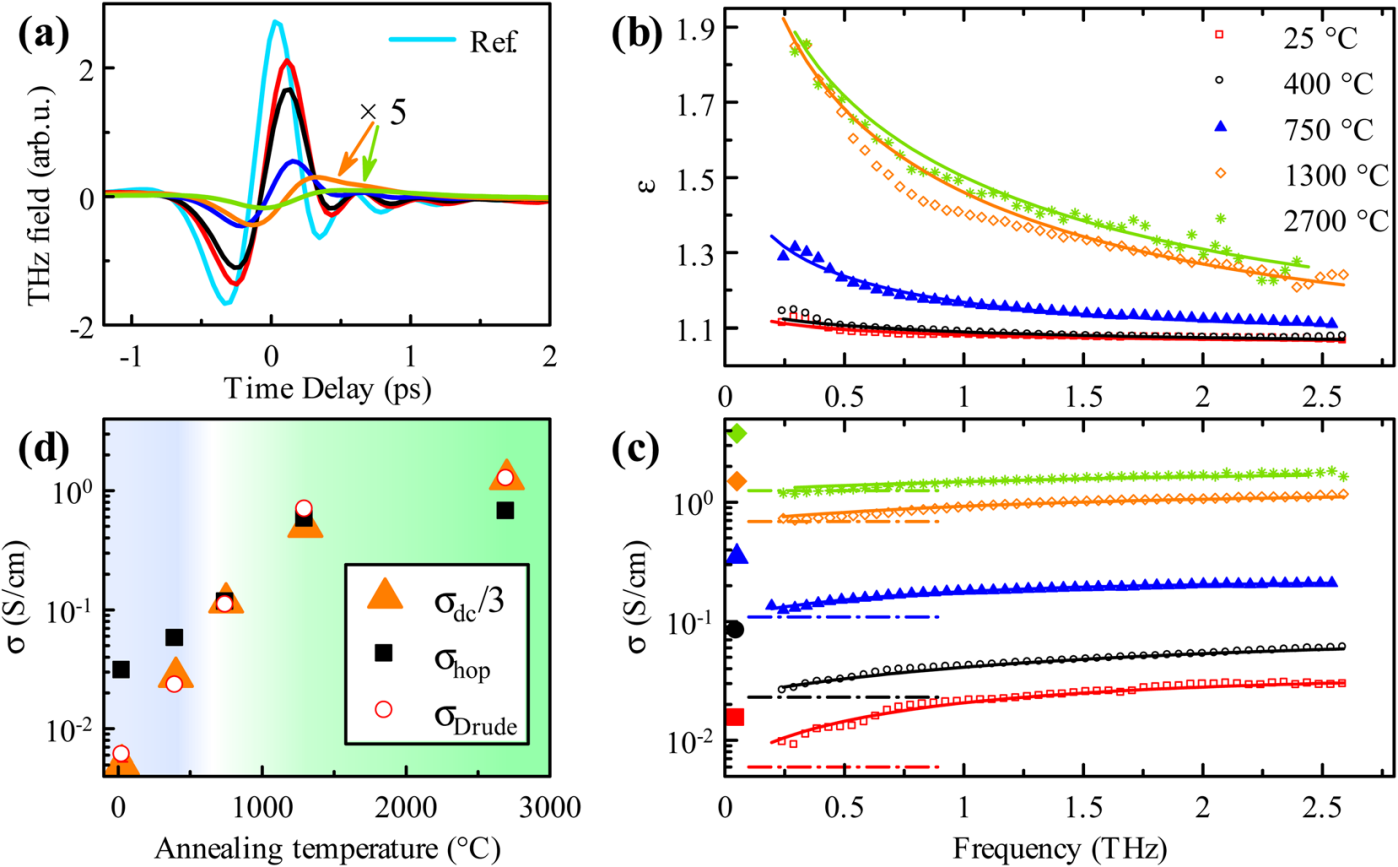
(a) Steady-state THz waveforms of GA samples with variable annealing temperature; color code is the same as in (b); waveforms for samples annealed at 1300 and 2700 °C are magnified 5 times to increase their visibility. (b) The real part of the permittivity, (c) the real part of the conductivity; symbols correspond to experimental data, and lines are fits of the hopping model given by eqn (2) and (3). Dash-dot lines indicate the magnitude of the Drude contribution *σ*_Drude_; larger symbols on the left-hand side of the plot correspond to dc measurements. (d) Hopping (*σ*_hop_) and Drude (*σ*_Drude_) contributions to the conductivity obtained from the fits. The blue background indicates dominant hopping, while the green background indicates dominant Drude conductivity. We plot here also *σ*_dc_/3 to emphasize qualitatively the same evolution of the Drude and dc conductivity components.

Room temperature THz spectra of the set of GA samples with different annealing temperatures are shown in Fig. 2. The data show an interesting feature of a simultaneous increase of the conductivity and a decrease of the permittivity with frequency. Such behaviour is strikingly different from the previously reported THz response of 2D layers of graphene.^5,38–40^ It is also incompatible with the response of confined carriers in the nanostructures^16,41^ and with localized plasmon resonance caused by depolarization fields, *e.g.*, in epitaxial graphene.^16,18^ Both these cases would imply an increasing permittivity with frequency, which is in sharp disagreement with the experimental observations. Clearly, the observed behaviour is rather a sign of a relaxation-like process. Similar THz ultrafast conductivity and permittivity spectra were previously obtained in amorphous and microcrystalline silicon.^42^ As these silicon materials contain numerous defects and grain boundaries, the conduction is realized through the hopping process of localized electrons and through band-like conduction of free electrons. Quite a similar situation can be encountered in the GA samples containing graphene and GO parts within the network of interconnected graphene sheets. In this situation, the band-like conductivity is expected to occur in the low-defect graphene regions, while the hopping process among localized electron states prevails in the more insulating GO parts with a larger density of defects.

We propose to describe the observed processes by the random free-energy barrier model proposed by Dyre.^43^ This model is one of the rare models that can describe a hopping conductivity even up to THz frequencies. Dyre's model captures the main features of the observed relaxational response in our experiment. It features lower and upper cut-off frequencies *ω*_min_ and *ω*_max_, respectively, which delimit the activation range of the hopping process and the conductivity dispersion. Since *ω*_min_ is typically several orders of magnitude below the THz frequency range, its influence in the THz spectra is irrelevant and the behavior can be described by an approximate formula.^42^ Altogether, the fitting formula reads:2
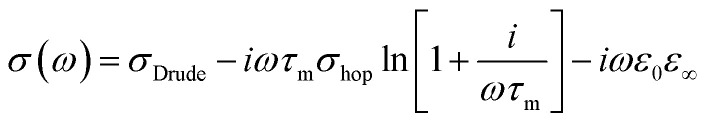
where *τ*_m_ = 1/*ω*_max_, *ε*_∞_ is the high-frequency permittivity of GA, *σ*_Drude_ is the contribution of conduction band carriers, and *σ*_hop_ is the saturated (high-frequency) hopping conductivity contribution. The Drude term is considered frequency independent in the accessible spectral range because the carrier dephasing time is very short, as confirmed below by the time-resolved THz measurements. The dispersion of the real part of the permittivity can be simply deduced:3
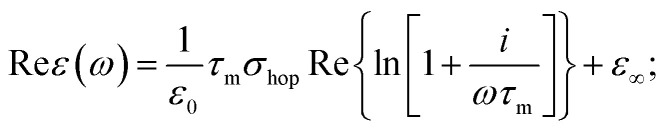


Note that the Drude term does not contribute to the real part of the permittivity in the case of the very short carrier scattering time: the whole observed dispersion is due to the relaxation mechanism (hopping). The fits of the experimental data using the model (2,3) are shown in Fig. 2b and c. All the values resulting from the fits are summarized in Table 1. The background permittivity *ε*_∞_ and the upper cut-off frequency *ω*_max_ do not show a clear trend with annealing temperature and are not important for further discussion of the physics of our materials. On the other hand, the individual contributions to the conductivity *σ*_Drude_ and *σ*_hop_ are fundamental; their behavior can be clearly seen in Fig. 2d. For the non-annealed GA sample, the hopping conductivity dominates, and the Drude contribution is negligible. The two contributions become of equal importance in the sample annealed at 750 °C. The Drude contribution increases by more than 2 orders of magnitude upon annealing at 2700 °C. Interestingly, the hopping process also increases its amplitude with increasing annealing temperature, meaning that more carriers are available in shallow states for the hopping. The saturation of the conductivity for high-temperature annealing at 2700 °C corroborates the stabilization of the composition of the highly annealed GA (Fig. 1e). As observed in Fig. 2d the dc conductivity exhibits the same trend *versus* annealing temperature as the Drude conductivity term obtained from the THz data, but their absolute values are systematically different (by about a factor of 3). The applied stress in dc measurements might have caused some change in the conductivity due to the induced strain in the samples,^25,26^ but it could not lead to a 3-fold increase. Note, however, that a precise quantitative comparison between the dc and THz conductivity is generally difficult in complex materials since different areas and conducting pathways are probed by the two techniques.

**Table 1:** The values of the *σ*_Drude_, *σ*_hop_, *ω*_max_ and *ε*_∞_ obtained by fitting the experimental data

Annealing temperature (°C)	*σ*_Drude_ [S m^−1^]	*σ*_hop_ [S cm^−1^]	*ω*_max_ [10^13^ s^−1^]	*ε*_∞_
Non annealed	0.60 ± 0.1	3.1 ± 0.1	1.6 ± 0.2	1.06 ± 0.01
400	2.3 ± 0.1	5.7 ± 0.2	2.5 ± 0.2	1.05 ± 0.01
750	10.9 ± 0.2	11.5 ± 0.2	1.1 ± 0.2	1.08 ± 0.01
1300	69 ± 1	57 ± 2	1.9 ± 0.2	1.07 ± 0.01
2700	125 ± 1	66 ± 2	2.3 ± 0.2	1.07 ± 0.01

**Fig. 3:**
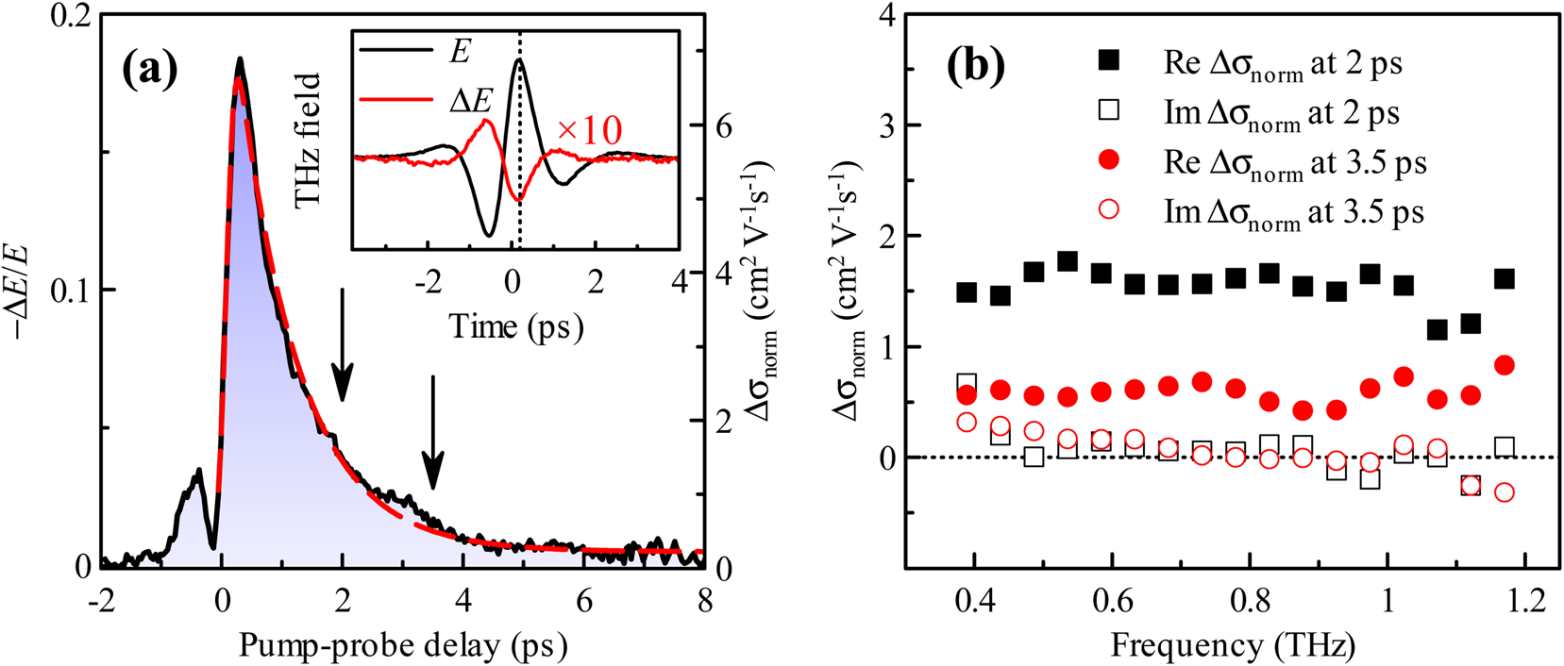
(a) Kinetics of the transient THz signal after photoexcitation (*ϕ* = 1.5 × 10^15^ photons per cm^2^) of a GA sample annealed at 2700 °C. Black solid line: experimental data; red dashed line: fit with a convolution of a single exponential and gaussian (instrumental) function. Inset: 10 times magnified transient waveform Δ*E* obtained at the pump-probe delay of 2 ps compared to the reference (steady-state) waveform *E*. (b) Spectra of complex photoconductivity per single unit charge Δ*σ*_norm_, see eqn (1), measured at 2 and 3.5 ps delays after photoexcitation, *cf.* arrows in (a).

The optical pump-THz probe measurements of GA samples exhibit very fast kinetics (see Fig. 3 for GA sample annealed at 2700 °C). A transient waveform (Δ*E*, measured at a pump-probe delay of 2 ps) and the equilibrium transmission waveform (*E*) are shown in the inset of Fig. 3a. The signal corresponds to photoinduced THz absorption (Δ*E* has an opposite sign than *E*). The kinetics in Fig. 3a is a pump-probe delay scan measured for the THz gated detection set at 0.2 ps (denoted by the dotted line in the inset). Thus, it can be considered as a time evolution of the amplitude of the waveform. A fit of the experimental data provides the photoconductivity lifetime of 1.0 ps. The origin of the pump-probe delay was carefully determined by a time-resolved measurement of the rise of the photoconductivity signal in a GaAs single crystal wafer inserted into the sample place. The small peak observed at the negative pump-probe delays in Fig. 3a is due to a re-shaping of the THz pulse as it encounters the onset of photoconductivity.^44^

The THz photoconductivity spectra evaluated following eqn (1) are shown in Fig. 3b. They are characterized by a nearly flat real part and a vanishing (within experimental errors) imaginary part. The time decay of the transient signal corresponds to the decay of the amplitude of the real photoconductivity. This spectral behavior is typical for a Drude-like contribution to the conductivity with a very short scattering time (≲20 fs) of the carriers. It corresponds to photoexcitation of free carriers into the conduction band and their picosecond trapping into hopping states or even into deeper localized states. These findings justify our assumption of the frequency-independent Drude contribution in the THz range used in the steady-state fits. The magnitude of the transient signal of the GA annealed at 1300 °C is similar to the GA sample annealed at 2700 °C. The signal decreases for samples annealed at lower temperatures (*e.g.*, an order of magnitude for the sample annealed at 400 °C). The lifetime of photocarriers in these low-temperature annealed samples is also somewhat shorter, ≲0.4 ps, and it is at the limit of the resolution of our experimental technique. This acceleration of the charge dynamics naturally corroborates a larger concentration of defects in these GA samples due to significantly more traps for the photocarriers.

If we assume that each absorbed pump photon generates a mobile charge, then the initial normalized transient conductivity directly represents the mobility of the photogenerated charges. The maximum value of the dynamics in Fig. 3 (reaching ∼7 cm^2^ V^−1^ s^−1^ for the GA sample annealed at 2700 °C) can then be understood as an order of magnitude estimate (in fact a lower limit, since not all absorbed photons may generate mobile carriers) of the charge carrier mobility in this sample. Assuming similar carrier mobility in the ground state and using the steady-state Drude conductivity value (1.25 S cm^−1^, Fig. 2d), we can make an order of magnitude estimation of the upper limit of the density of free carriers in the ground state. We find the charge carrier density of *n* ≲ 10^13^ cm^−2^ for the sample annealed at 2700 °C by estimating the volume filling fraction of carbon in the GA sample (∼0.3%) using the ratio between the mass density of our GA samples (6–7mg cm^−3^) and pristine graphene (2.3 g cm^−3^).^45^ It should be noted that the mobility is very low compared to standard 2D graphene layers,^46^ suggesting that there is ample space for improvement.

The measured steady-state and transient effective conductivity spectra reflect the morphology and particularly the percolation degree of materials.^31^ The character of the measured spectra indicates that the investigated graphene sheets form a network with a high degree of percolation.

**Fig. 4:**
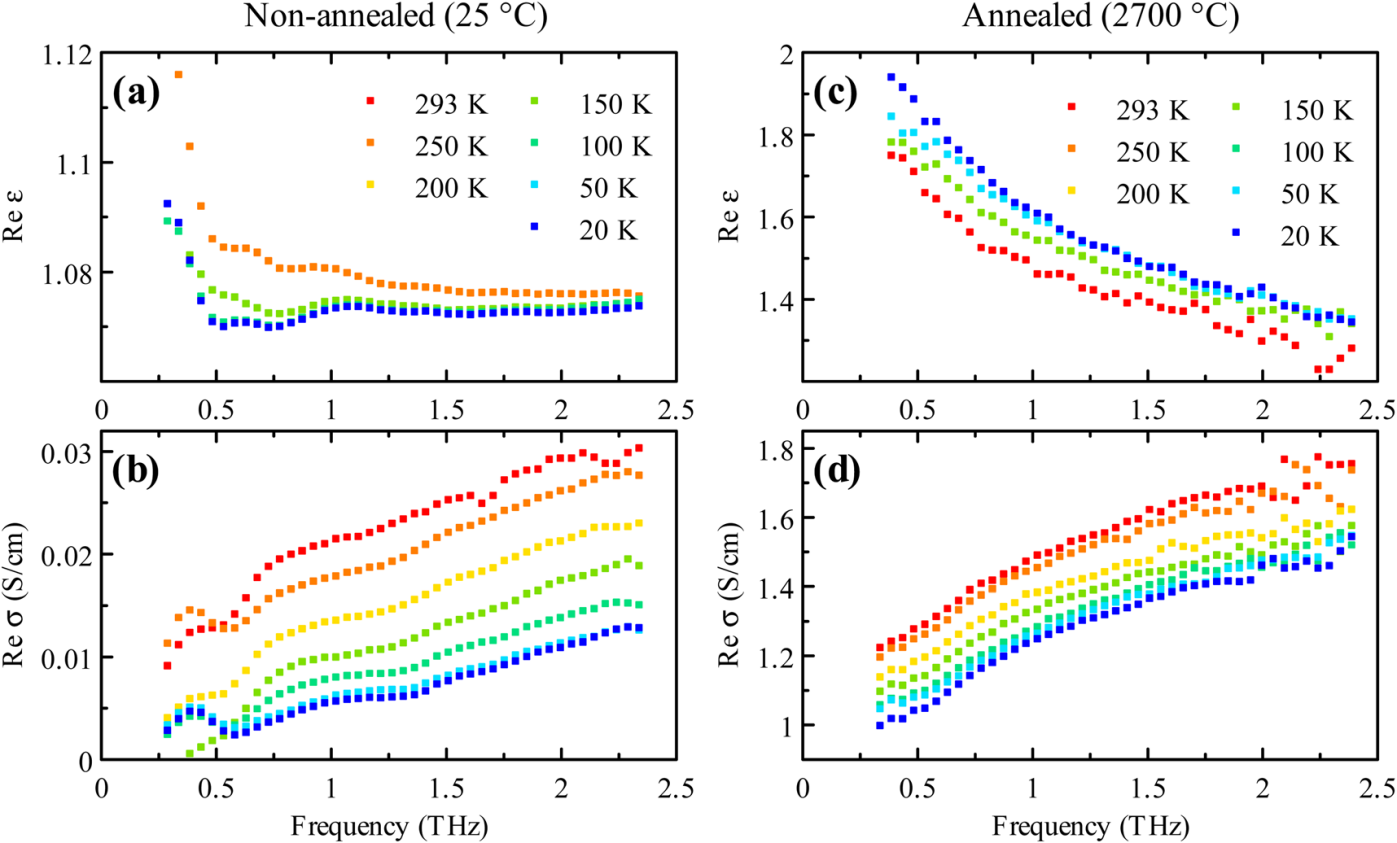
(a and b) Temperature dependence of the real parts of the permittivity and conductivity in the steady-state of the non-annealed sample. (c and d) Temperature dependence of the real parts of the permittivity and conductivity in the steady-state of the sample annealed at 2700 °C. Only selected temperatures are shown for Re*ε* since its overall temperature variation is very small.

We also investigated the steady-state spectra of the samples as a function of temperature. Fig. 4 shows the conductivity and permittivity spectra measured for the non-annealed sample (Fig. 4a and b) and the sample annealed at 2700 °C (Fig. 4c and d). The spectra do not exhibit any qualitative change of the behavior upon the temperature variation. The conductivity slightly and monotonously decreases with the decreasing temperature. The dc conductivity has also shown a similar trend (not shown here). The permittivity also does not reveal any significant change with temperature, which suggests no modification of the nature of carrier transport in the samples. The decrease in the conductivity can be attributed to a reduction in the concentration of the charge carriers, which are thermally released from the traps at high temperatures. In this view, the steady-state Drude contribution decreases at low temperatures. Note that the conductivity of the non-annealed sample tends to zero at low temperatures and low frequencies. The behavior of other studied samples annealed at different temperatures does not differ qualitatively from what is shown in Fig. 4. The room temperature spectra and temperature dependence of the permittivity and conductivity of these samples are similar to the spectra shown in Fig. 2 and 4, respectively.

## Conclusion

This work represents a systematic investigation of the charge carrier dynamics in the 3D structure of graphene aerogels. The charge transport mechanism exhibits both intraband transitions and hopping transport modes at THz frequencies. This behavior is strikingly different from that observed in 2D graphene. The hopping process controls the transport in defect-rich parts of the 3D graphene networks. Conversely, in parts with low defect concentration, a Drude band-like conductivity with very short carrier scattering time takes over the transport mechanism. Higher annealing temperatures lead to an increase in the Drude conductivity and, to a lesser extent, in the high-frequency hopping contribution due to the presence of shallow states. Free carrier density in the sample annealed at 2700 °C reaches 10^13^ cm^−2^ while their mobility is very low (of the order of 10 cm^2^ V^−1^ s^−1^), thus indicating a large space for improvement. Optical pump-THz probe results reveal Drude photoconductivity with very fast decay (∼1 ps). Our study opens new prospects for understanding charge transport in multidimensional structures made up of 2D layers.

## Conflicts of interest

The authors declare no conflict of interest.

## Supplementary Material
